# Dexamethasone inhibits IL-9 production by human T cells

**DOI:** 10.1186/1476-9255-2-3

**Published:** 2005-04-20

**Authors:** Lauren E Holz, Kristoffer P Jakobsen, Jacques Van Snick, Francoise Cormont, William A Sewell

**Affiliations:** 1 Garvan Institute of Medical Research, 384 Victoria St, Darlinghurst, NSW 2010, Australia; 2 Centre for Immunology, St. Vincent's Hospital, University of NSW, NSW 2052, Australia; 3 Ludwig Institute of Cancer Research, Brussels Branch and the Experimental Medicine Unit, Universite de Louvain, B-1200 Brussels, Belgium; 4 St Vincent's Clinical School, University of NSW, NSW 2052, Australia

## Abstract

**Background:**

Interleukin 9 (IL-9) is produced by activated CD4+ T cells. Its effects include stimulation of mucus production, enhanced mast cell proliferation, enhanced eosinophil function, and IgE production. These effects are consistent with a role in allergic diseases. Glucocorticoids have potent anti-inflammatory effects, including suppression of cytokine synthesis, and are widely used in the treatment of allergic conditions.

**Methods:**

We examined the effect of the glucocorticoid dexamethasone (Dex) on IL-9 mRNA expression and protein secretion with real-time RT-PCR and ELISA. Peripheral blood mononuclear cells (PBMC) were prepared from human volunteers and activated with OKT3. CD4+ T cells were purified from PBMC and activated with OKT3 plus PMA.

**Results:**

IL-9 mRNA abundance and protein secretion were both markedly reduced following treatment of activated PBMC with Dex. mRNA levels were reduced to 0.7% of control values and protein secretion was reduced to 2.8% of controls. In CD4+ T cells, Dex reduced protein secretion to a similar extent. The IC_50 _value of Dex on mRNA expression was 4 nM.

**Conclusion:**

These results indicate that IL-9 production is very markedly inhibited by Dex. The findings raise the possibility that the beneficial effects of glucocorticoids in the treatment of allergic diseases are in part mediated by inhibition of IL-9 production.

## Background

CD4+ T cells of the T helper 2 (Th2) type have been implicated as major contributors to the pathology of allergic asthma [1]. Th2 cells produce the cytokines IL-4, IL-5, IL-9 and IL-13. IL-9, which was first identified as a T cell growth factor [2], has multiple effects consistent with a role in allergic inflammation. IL-9 acts on the pulmonary epithelium to induce production of mucus [3] and chemokines [4]. It enhances eosinophil function via induction of the IL-5 receptor [5]. IL-9 induces immunoglobulin synthesis of all isotypes, especially IgE [6]. Mast cell numbers are elevated in the lung by IL-9 [7].

There is evidence in clinical studies for an association between IL-9 and allergic asthma. In bronchial biopsies, the cells expressing IL-9, which were predominantly T cells, were increased in patients with allergic asthma, and this was associated with bronchial hyper-reactivity [8,9]. An association between IL-9 expressing cells and eosinophilia has also been described [10]. In allergic asthma patients, IL-9 in the bronchoalveolar fluid was increased after segmental allergen challenge [11]. Among a range of cytokines produced by *in vitro *stimulated PBMC, IL-9 was found to have the best correlation with allergic reactivity as measured by skin prick tests [12].

Several animal studies have investigated the role of IL-9 in allergic asthma. In transgenic mice with elevated pulmonary expression of IL-9, there was increased influx of inflammatory cells to the lungs, increased mucus production and increased mast cell numbers [13]. In two separate mouse model studies of allergen-induced asthma, administration of neutralising anti-IL-9 antibodies reduced eosinophilia, BHR, airway damage and IgE [14,15]. In a model of parasitic infection with a Th2 response, IL-9 knockout mice displayed markedly reduced goblet cell hyperplasia and mastocytosis [16]. However, in a model of allergic asthma, airway hyperreactivity, eosinophilia and goblet cell hyperplasia were not impaired in IL-9 knock-out mice [17]. Despite the findings in knock-out mice, overall the evidence from animal models is consistent with clinical evidence that IL-9 may have a role in allergic asthma.

Glucocorticoids (GC) are a major component of the treatment of asthma and other allergic disorders. GC bind to cytoplasmic glucocorticoid receptors (GR) and GC/GR complexes translocate to the cell nucleus where they stimulate or inhibit the transcription of a large number of genes. The anti-inflammatory effects of GC have been associated with inhibition of transcription of numerous cytokines [18]. GC markedly reduce gene transcription of the Th2 cytokines IL-4 [19], IL-5 [20] and IL-13 [21] as well as inhibiting the production of many other cytokines including IL-2 [22], GM-CSF [23] and interferon gamma (IFN-γ) [24]. By contrast, GC induce the expression of certain cytokines, including IL-10 [25], IL-1 receptor antagonist (IL-1Ra) [26] and transforming growth factor-beta [27], and GC do not affect expression of M-CSF [23].

Given the extensive evidence indicating IL-9 may be a candidate cytokine in the pathogenesis of allergic diseases, further research into the regulation of IL-9 production is warranted. Because glucocorticoids are effective in the treatment of allergic diseases, it is important to understand their effects on genes that are potentially relevant to the pathogenesis of these diseases. Therefore we have investigated the effect of the synthetic glucocorticoid dexamethasone (Dex) on IL-9 production.

## Methods

### Cell culture

Peripheral blood was donated by healthy volunteers from the Garvan Institute of Medical Research and the Centre for Immunology. The procedures were approved by the Human Research Ethics Committee, St Vincent's Hospital, Sydney and are in compliance with the Helsinki Declaration. Peripheral blood mononuclear cells (PBMC) were isolated by ficoll-based density centrifugation. Cells were resuspended in complete medium consisting of RPMI 1640 medium (JRH Biosciences, Lenexa, KS, USA) supplemented with 10% v/v heat-inactivated foetal bovine serum (FBS) (CSL Ltd, Parkville, Australia), 2 mM L-glutamine, 20 mM HEPES buffer, 100 U/mL penicillin and 100 μg/mL streptomycin (all from Invitrogen, Carlsbad, CA, USA). Cell counts and viabilities were determined by trypan blue exclusion in a haemocytometer. Viability was always greater than 95%.

PBMC were adjusted to 1 × 10^6 ^cells/mL and were incubated at 37°C in 5% CO_2 _for the activation period. PBMC were treated with 100 ng/mL OKT3 (diluted in PBS) or with a corresponding volume of PBS. OKT3, a kind gift of Janssen-Cilag, Sydney, Australia, causes T cell activation by binding to the T-cell specific surface molecule CD3. Cells were treated with Dex (Sigma, Castle Hill, Australia) or with a corresponding volume of PBS. Dex was diluted in PBS and added immediately after OKT3.

In some experiments, CD4+ T cells were purified by incubating PBMC in complete medium for 90 minutes at 37°C and 5% CO_2 _to deplete adherent cells. The non-adherent cells were then centrifuged and resuspended in MACS Buffer (0.5% FBS and 2 mM EDTA in PBS) and MACS human CD4^+ ^micro beads (Miltenyi Biotec, Auburn, California, USA) according to the manufacturer's instructions. After incubation, the cells were washed and CD4^+ ^cells were then isolated by a MACS LS Column placed in a MACS Separator according to the manufacturer's instructions (Miltenyi).

Small aliquots of the CD4^+ ^cells were analysed by flow cytometry. Cells were stained with anti-CD3 FITC and anti-CD4 PE antibodies and analysed on a FACSCalibur using CellQuest software (all BD Biosciences, San Jose, CA). At least 98% of the cells expressed CD3 and CD4. CD4^+ ^cells were cultured as above except that they were stimulated with a combination of 8 ng/mL PMA (Sigma) and plate-bound OKT3. OKT3 was bound to 12-well plates by addition of 10 μg/mL of OKT3 in PBS at 4°C overnight. The antibody solution was removed immediately prior to addition of the cells.

### RT-PCR

After culture for 24 hours, cells were centrifuged at 440 *g *for 5 min. Total RNA was extracted by Trizol (Invitrogen) according to the manufacturer's instructions. RNA was dissolved in DEPC-treated water and stored at -70°C until required. RNA concentration was determined by spectrophotometry. 2 μg of total RNA was heated to 65°C for 5 min, cooled for 2–3 min on ice, and reverse transcribed by avian myeloblastosis virus reverse transcriptase (AMV-RT), with 1 μM oligo (dT)_15 _primer (Roche, Castle Hill, Australia), 20U AMV-RT enzyme (Roche), 1 mM dNTP (Roche), AMV-RT buffer (50 mM Tris-HCl, 8 mM MgCl_2_, 30 mM KCl, 1 mM dithiothreitol) (Roche) and DEPC-water in a 20 μL volume at 42°C for 1 hour. Tubes were heated to 65°C for 5 min and stored at -20°C until required.

For IL-9 PCR, in a 20 μL reaction mixture, 1 μL cDNA was amplified by Platinum Quantitative PCR Supermix UDG (1.5 U Platinum Taq Polymerase, 20 mM Tris-HCl, 50 mM KCl, 3 mM MgCl_2_, 200 μM dGTP, 200 μM dATP, 200 μM dCTP, 200 μM dUTP, 1U Uracil DNA glycosylase (UDG)) (Invitrogen), Milli-Q water, 0.4 μM of the forward and reverse primers and Taqman probe (Geneworks, Rundle Mall, Adelaide, Australia) with sequences 5'CCTGGACATCAACTTCCTCATC3', 5'CATGGCTGTTCACAGGAAAA3' and 5'FAM-CTCTGACAACTGCACCAGA-TAMRA3', respectively. PCR was performed with a Rotorgene 3000 real-time PCR machine (Corbett Research, Mortlake, Sydney, Australia). No template controls (NTC) with water instead of cDNA were included in all experiments. The reaction conditions for the IL-9 real-time PCR were 95°C for 3 min followed by 40 cycles of 95°C for 15 sec then 60°C for 60 sec. Forward and reverse primers were designed to bind to different exons so that any genomic DNA amplification could be distinguished from cDNA.

The PCR amplification efficiency was determined in every experiment by serial four-fold dilutions of the activated sample containing no Dex. These diluted samples and all the undiluted samples were analysed again in duplicate by real-time PCR under the same conditions. The amplification efficiency was determined by plotting the mean threshold cycle (Ct) value of the diluted samples against the log of the dilution. IL-9 amplification efficiencies ranged from 1.63 to 1.99. The actual amplification efficiencies were then used to determine the ratios of samples treated with and without Dex.

A β-actin PCR was also performed on each sample. 1 μL cDNA was amplified in 25 μL in PCR buffer (10 mM Tris-HCl, 1.5 mM MgCl_2_, 50 mM KCl) (Roche), 0.25 mM dNTP (Roche), 1 X SybGr (Molecular Probes, Eugene, OR, USA), 0.75 U Taq polymerase (Roche), 2 mM MgCl_2 _and 0.32 μM of forward and reverse β-actin primer (Geneworks) with sequences 5'CCAACTGGGACGACATG3' and 5'CAGGGATAGCACAGCCT3' respectively [20]. Samples were amplified by 94°C for 2 min followed by 30 cycles of 94°C for 15 sec, 56°C for 20 sec and 72°C for 20 sec. To confirm the identity of PCR products, all products were size-fractionated by agarose gel electrophoresis, and products with apparent mobility consistent with the expected size (277 bp for IL-9 and 203 bp for β-actin) were detected.

### ELISA assays

ELISA assays were used to determine the IL-4, IL-9 and IFN-γ concentration in the culture supernatants. The IL-9 reagents (capture antibody, standard, and detection antibody) have been described previously [28], whereas the IL-4 and IFN-γ kits were purchased from BD Biosciences. 384 well flat bottom MAXISorp plates (Nunc, Roskilde, Denmark) were used. In the IL-9 ELISA the capture antibody, mh9a4, was diluted in a coating buffer (20 mM glycine, 30 mM NaCl, pH 9.2) at a concentration of 5 μg/mL. After overnight incubation at 4°C and washing with 0.05% Tween-20 in PBS, the plate was blocked with the assay diluent, 1% (w/v) BSA in PBS, incubated at 37°C for at least 2 hours and washed again. Before a final overnight incubation at 4°C the samples and standards were prepared in assay diluent, and loaded into the wells in triplicate. The standards were prepared in two-fold dilutions from 500 pg/mL to 3.9 pg/mL. After washing, detection antibody mh9a3-biotin was added in a 1:2000 dilution for 2 hours at 37°C. The plates were washed and streptavidin-horseradish peroxidase conjugate was added (DakoCytomation, Glostrup, Denmark) 1:500 in assay diluent, and plates were incubated at room temperature for 30 min. The IL-4 and IFN-γ ELISA assays were performed according to the manufacturer's instructions. The lower limits of detection were 3.9–15.6 pg/mL for IL-9, 7.8 pg/mL for IL-4 and 3.9 pg/mL for IFN-γ. When results with and without Dex were presented as percentages, if a sample was undetectable in the ELISA, the lower limit of detection of the assay was used in the calculation.

All assays were washed, loaded with TMB Substrate solution (BD Biosciences) in a 1:1 mixture of TMB substrate A and B, and incubated at room temperature in the dark for 30–45 minutes before the reaction was stopped with 2 M H_2_SO_4_. Absorbance was measured by a Spectra Image reader using X-read Plus software (both Tecan, Maennedorf, Switzerland).

### Statistics

Samples were compared with the Wilcoxon signed rank test (Statview Software 5.0, Abacus Concepts, Berkeley, California, USA). A *p *value of <0.05 was considered significant.

## Results

### Dexamethasone reduces IL-9 mRNA abundance

**Figure 1:**
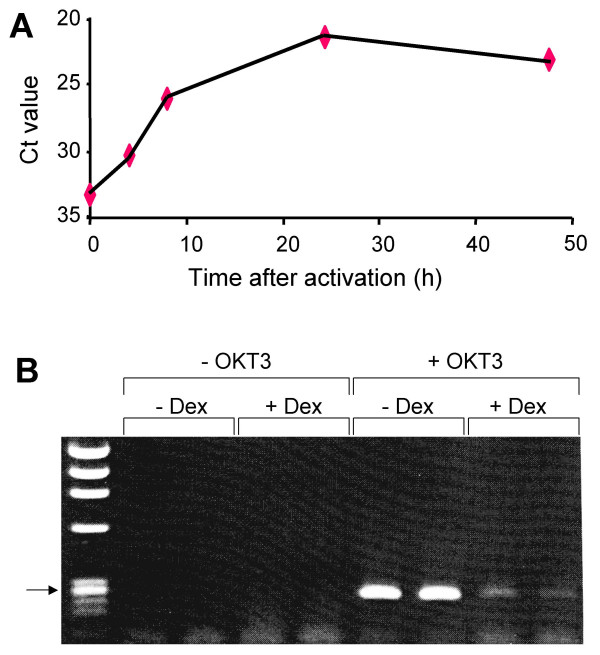
**IL-9 RT-PCR. **A. Time course. PBMC were incubated for various times with OKT3, RNA was extracted, IL-9 real time RT-PCR was performed, and the mean threshold cycle (Ct) was determined. The data shown are from an experiment on one representative individual. The values are means of duplicate determinations. B. Gel electrophoresis. PBMC were incubated for 24 h with or without OKT3 and with or without Dex (10-6 M). RNA was extracted and IL-9 RT-PCR performed for 40 cycles. For each condition, duplicate PCRs were performed on cDNA from one representative individual. Products were analysed in a 2% agarose gel. The left lane contains HaeIII cut ΦX174 molecular size markers (Roche); the arrow indicates the position of the 281/271 bp markers.

In preliminary experiments, real-time RT-PCR revealed that OKT3 was a highly effective stimulus of IL-9 expression in PBMC, as previously reported [2]. IL-9 mRNA was induced from 4 to 48 h after activation (Fig. 1A), and 24 h was chosen as a suitable time for detection of mRNA in subsequent experiments. The effect of Dex on IL-9 mRNA abundance in PBMC was examined in 13 healthy individuals by real-time RT-PCR. PBMC were cultured with OKT3, with or without 10^-6 ^M Dex. In all samples treated with OKT3 without Dex, IL-9 mRNA expression was readily detected. Addition of Dex to cultures stimulated by OKT3 was followed by a marked reduction in IL-9 mRNA abundance. All samples treated with Dex had much higher Ct values than those without Dex (Table 1). Statistical analysis revealed a highly significant effect of Dex (*p *< 0.01). All RT-PCR products were subjected to gel electrophoresis and the results were consistent with the real-time data. In the samples activated with OKT3, a single strong band was detected with apparent mobility consistent with the predicted fragment size of 277 bp (Fig. 1B). After treatment with OKT3 and Dex, a very faint band of the same mobility was detected, and no bands were detected in the unactivated samples. Except for some very low molecular size material, there was no evidence of any other band apart from the 277 bp band. The cDNA samples were also assessed for the housekeeping gene β-actin by real-time RT-PCR, and Dex had no significant effect. In activated cells, Ct values for β-actin were 15.5 ± 2.7 (SD) for samples given Dex, compared with 16.5 ± 4.6 for samples not given Dex. The findings with β-actin indicate that Dex did not cause a generalized reduction of gene expression.

**Table 1:** Effect of Dex on IL-9 mRNA in 13 different individuals.

Expt	Ct no Dex	Ct with Dex	Amplification Efficiency	% IL-9 in Dex vs no Dex
1	22.6	34.3	1.74	0.15
2	21.1	32.1	1.76	0.20
3	24.0	35.0	1.91	0.08
4	21.6	30.8	1.85	0.34
5	27.0	35.8	1.70	0.91
6	23.1	34.6	1.79	0.12
7	20.9	37.8	1.85	0.03
8	24.5	33.7	1.85	0.34
9	22.0	30.3	1.63	1.72
10	24.5	30.2	1.79	3.57
11	19.7	32.2	1.70	0.13
12	24.0	30.7	1.99	1.00
13	23.2	34.6	1.79	0.13

PBMC were activated with or without 10^-6 ^M Dex, and Ct values for IL-9 were determined. The amplification efficiencies were measured for each sample, and were applied to the Ct differences between the Dex and no Dex samples to determine the proportion of IL-9 in samples treated with and without Dex.

The relative change in IL-9 mRNA expression produced by Dex was ascertained by calculating the difference in Ct values between the activated and activated + Dex samples (Table 1). This difference was then corrected for the amplification efficiency of samples from each individual PBMC donor. Amplification efficiency was determined by serial dilution of each of the samples activated and not treated with Dex. The percentage of IL-9 transcription in the Dex-treated samples compared to controls ranged from 0.03% to 3.57% with a mean of 0.67% and a median of 0.20%.

### Concentration-response studies

**Figure 2:**
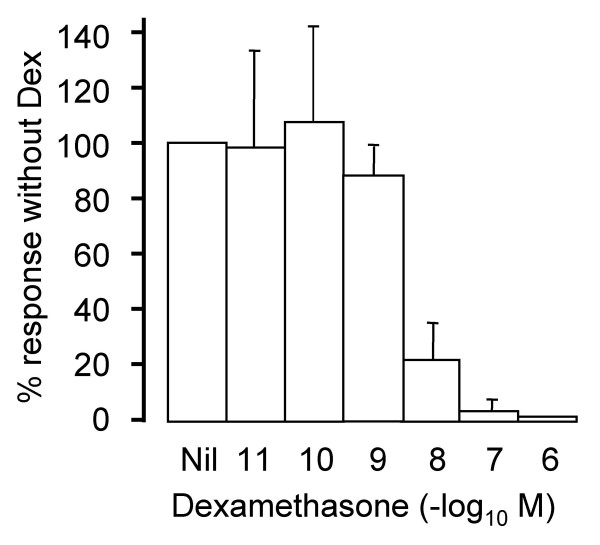
**Concentration-response effect of Dex on IL-9 mRNA in activated PBMC. **Cells were incubated with OKT3 and the stated concentration of Dex. 24 hours later, RNA was extracted and real time RT-PCR for IL-9 was performed. Data were corrected for amplification efficiency as described in Methods. Each sample was measured in duplicate. The results are expressed as the % of the response in cells not treated with Dex. The data are the mean ± SEM of four different individuals.

The effectiveness of Dex was assessed by comparing IL-9 transcription in samples not treated with Dex to samples treated with 10^-6 ^M to 10^-11^M Dex. Mean Ct values of duplicate samples were determined, and in each individual the mean Ct value of the sample not treated with Dex was given a figure of 100%. PCR was then performed on serial dilutions of the samples not treated with Dex to correct for amplification efficiency as described in the Methods. Dex inhibited IL-9 transcription in PBMC activated with OKT3 in a concentration dependent manner in four different individuals. The average percentage value for each Dex concentration is plotted in Figure 2. 10^-7^M Dex was almost as inhibitory as 10^-6 ^M Dex, and 10^-8 ^M Dex reduced IL-9 transcript abundance to 20% of control levels. At lower concentrations of Dex, transcription increased towards control levels. In 2 of 4 experiments, the samples treated with 10^-10 ^M Dex had a higher level of transcription than control samples, contributing to the slightly higher average IL-9 expression level at 10^-10 ^M Dex compared with no Dex (Fig. 2). The concentration of Dex that inhibited 50% of IL-9 transcription in activated PBMC, the IC_50_, was calculated to be 10^-8.4 ^M or 4 nM.

### Dex inhibits IL-9 protein secretion

**Figure 3:**
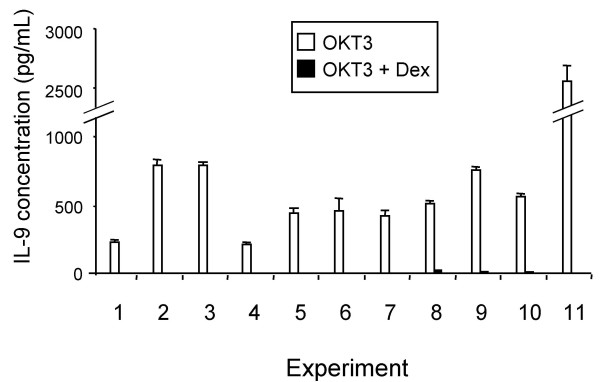
**Effect of Dex on IL-9 secretion by PBMC. **Cells from 11 different individuals were treated with OKT3 and with or without 10^-6 ^M Dex. Culture supernatants were harvested 48 hours later and measured for IL-9 by sandwich ELISA. Data represent the mean ± SD of triplicate determinations.

After activation with OKT3, IL-9 secretion was readily detected by sandwich ELISA. Supernatants were harvested at various times after activation, and IL-9 was measured in triplicate. The amount of IL-9 after 72 h of culture was defined as 100%. IL-9 was not detected at 0 h. At 24 h, the IL-9 level was 16 ± 1 % (mean ± SD) and at 48 h it was 85 ± 3 %. Thus IL-9 levels had almost peaked by 48 h, and supernatants were harvested at this time in subsequent experiments. PBMC from 11 different donors were treated with or without OKT3 and with or without 10^-6 ^M Dex throughout the culture period. In all samples stimulated with OKT3, there were high levels of IL-9 secretion in the absence of Dex, and IL-9 concentrations were in the range of 207–2,526 pg/mL. IL-9 secretion was very markedly reduced after treatment with Dex (*p *< 0.005). In the Dex-treated samples, secretion was only 2.8 % ± 2.5% (SD) of control values (Fig. 3). In 8 of the 11 samples treated with OKT3 and Dex, the IL-9 concentration was below the lower limit of detection of the assay. In most of the cultures not treated with OKT3, IL-9 could not be detected. It was detected at very low levels in 3 samples in the absence of Dex and in 1 sample in the presence of Dex.

In six of the 11 samples, the culture supernatants were also tested for IFN-γ and IL-4. In activated cells treated with Dex, the IFN-γ and IL-4 concentrations were always above the lower detection limit of the assays. Dex significantly reduced the concentrations of both cytokines (*p *< 0.05 in both cases). The effect of Dex on IFN-γ secretion was similar to that on IL-9. Activated cells treated with Dex secreted 2.4 ± 2.1 % as much IFN-γ as control activated cells. By contrast, Dex had substantially less inhibitory effect on IL-4 secretion. The Dex-treated cells secreted 31.4 ± 14.1 (SD) % as much IL-4 compared with control activated cells.

**Figure 4:**
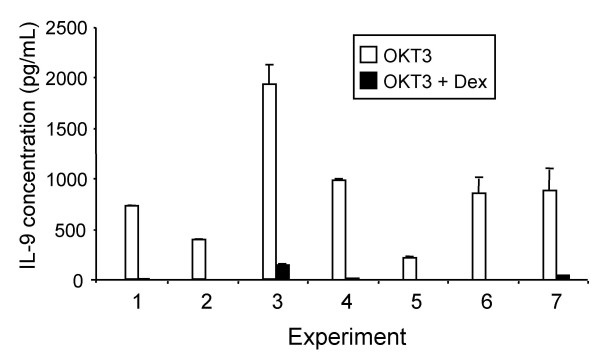
**Effect of Dex on IL-9 secretion by CD4+ T cells. **Cells from 7 different individuals were treated with OKT3 and with or without 10^-6 ^M Dex. Culture supernatants were harvested 48 hours later and measured for IL-9 by sandwich ELISA. Data represent the mean ± SD of triplicate determinations.

CD4+ T cells were purified from 7 individuals, to determine whether Dex was acting directly on these cells. In cells not activated with OKT3, IL-9 was detected at very low levels in 4 samples without Dex and in 2 samples in the presence of Dex. Activated cells not treated with Dex secreted IL-9 in the range 222–1,939 pg/mL. As with PBMC, Dex markedly inhibited IL-9 secretion in activated cells (Fig. 4) (*p *< 0.02). Samples treated with 10^-6 ^M Dex secreted only 2.9 % ± 2.5% (SD) as much IL-9 as control samples. In activated cells treated with Dex, IL-9 was below the detection limit of the assay in 3 of 7 cultures in these experiments.

## Discussion

The study demonstrates that Dex is an efficient pharmaceutical agent for inhibition of IL-9 production. In activated PBMC, Dex reduced IL-9 secretion to a mean of 2.8% of control levels, whereas in the case of mRNA, the corresponding value was 0.7 %. The difference between these 2 percentage values may have arisen because in the real-time PCR analysis, it was always possible to determine a value for IL-9 mRNA in the Dex-treated samples, whereas in the ELISA assay, the corresponding samples were usually undetectable. In the latter samples, the lower limit of detection of the assay was used to calculate percentages, which may have over-estimated the IL-9 concentration in the Dex-treated samples.

To determine if the inhibitory effect was specific for helper T cells, experiments were also carried out with purified CD4^+ ^cells. These populations contained at least 98% CD3+CD4+ cells, making it very likely that the observed effects directly involve helper T cells. The data indicate that CD4+ T cells produce substantial amounts of IL-9, although the possibility that other cells in PBMC also produce IL-9 has not been excluded. Dex markedly reduced IL-9 secretion in CD4^+ ^T cells, and the data are most consistent with a direct effect of Dex on CD4+ T cells.

Dex was found to inhibit the synthesis of IL-9 mRNA in PBMC in a concentration dependent manner. Marked inhibition of IL-9 transcription was observed with Dex concentrations as low as 10^-8 ^M, and Dex had an IC_50 _value of 4 nM. Similar Dex concentration response curves have been observed with IL-2 [22] and IL-5 [20] expression in T cells, as well as IL-4 and IL-5 in mast cells [29]. ICAM-1 expression [30] as well as prostaglandin synthesis and release in alveolar tissue [31] have also been found to have similar responses to a range of concentrations of Dex. IC_50 _values for Dex have been obtained for ICAM-1 expression of <1 nM [30], COX activity of 1–10 nM [31], IL-11 expression of 1 nM [32] and IL-5 expression in T cells of 1 nM [20]. In mast cells, Dex had an IC_50 _value of 1.6 nM on IL-5 expression indicating that the sensitivity of T cells and mast cells to Dex is similar for Th2 cytokines [29]. These findings, taken together, suggest that Dex may be inhibiting similar pathways involved in regulation of expression of a variety of different genes in T cells and mast cells.

Glucocorticoids can mediate effects on transcription in two ways. After translocation of the GC/GR to the nucleus, the GR can bind directly to glucocorticoid response element (GRE) sequences in the promoter regions of target genes. The expression of many genes is stimulated in this fashion. However there is limited evidence for GRE involved in inhibition of gene expression. Alternatively, GC act indirectly by GR binding to transcription factors so as to prevent them from interacting with DNA. Previous studies have found that the two mechanisms are mediated by different concentrations of Dex. The inhibitory effect of Dex on collagenase expression was found to be mediated by interaction between GC/GR and the transcription factor AP-1 [33]. In the absence of GC, AP-1 binds to the promoter of the collagenase gene to stimulate transcription, whereas in the presence of GC, binding between GC/GR and AP-1 prevents the latter from associating with DNA, so that transcription is inhibited. Half maximal repression of collagenase expression was reached with 1.5 nM Dex, whereas half-maximal induction of gene expression via GRE binding required 10 nM or greater [33]. We found Dex to have an IC_50 _value of 4 nM, consistent with an indirect effect via interference with transcription factor(s).

Among possible transcription factors, NF-AT is a likely candidate. In the case of the IL-5 promoter, we observed that Dex inhibited binding to the NF-AT site but not to the GATA-3 site [34]. The IL-9 promoter contains binding sequences for NF-AT [35], and the transcription of other cytokines including IL-2 [36] and IL-4 [37] involves NF-AT. IL-4, IL-5 and IL-9 all reside within the Th2 gene cluster on human chromosome 5 [38] raising the possibility that they may have similar regulatory mechanisms. Other factors which may be involved include AP-1, NF-κB and CREB, which have DNA binding sites in the IL-9 promoter [35] and which can be inhibited by glucocorticoids [36,39,40].

Expression of IL-9 by T cells may depend on the effects of other cytokines produced after activation [41]. This is consistent with the delayed induction of IL-9 mRNA, which did not peak until 24 h after activation (Fig. 1A). It is therefore possible that the effect of Dex on IL-9 production may be a consequence of its inhibitory effect on cytokines produced earlier after T cell activation. Dex inhibited the production of the key Th1 cytokine IFN-γ to a similar extent to IL-9 (Table 2). In other experiments on PBMC, we observed that 10^-6 ^M Dex reduced the secretion of IL-5 to 0.8 % of control PHA activated cells, and that of IL-13 to 6.2 % of controls (n = 6 for IL-5 and IL-13) (M. Irvine & W. A. Sewell, unpublished observations). However, not all Th2 cytokines are as markedly inhibited by Dex, because IL-4 was only inhibited to 31% of control levels (Table 2). The relative resistance of IL-4 to the inhibitory effects of Dex may explain an unexpected effect of Dex in enhancing the development of Th2 cells [42]; these findings could be explained by more efficient suppression by Dex of IFN-γ than IL-4, leaving sufficient IL-4 to favour differentiation of T cells into Th2 cells.

**Table 2:** Effect of Dex on IFN-γ, IL-4 and IL-9 secretion.

Cytokine	OKT3 range	OKT3 plus Dex range	% cytokine in Dex vs no Dex
IFN-γ (ng/mL)	11–56	0.15–1.1	2.4 ± 2.1
IL-4 (pg/mL)	23–81	12–22	31 ± 14
§ IL-9 (pg/mL)	234–781	* undetectable	4.3 ± 2.9

PBMC from 6 different individuals were activated with OKT3 and treated with or without 10^-6 ^M Dex. Cytokine concentration was measured in triplicate. For each individual, the % cytokine secretion in Dex versus no Dex was determined, and the Table shows the mean ± SD of these values. § The IL-9 data are for these 6 individuals only; the results are not significantly different from the results for all 11 individuals shown in Fig. 3. * For IL-9, all the Dex treated samples were below the lower limit of detection of the assay which was 7.8–15.8 pg/mL. The latter figures were used to calculate the % cytokine figure.

## Conclusion

IL-9 mRNA expression and protein secretion were very markedly inhibited by Dex. The findings suggest that the beneficial effects of glucocorticoids in the treatment of allergic diseases may, in part, be mediated by inhibition of IL-9 production. Glucocorticoids are a mainstay in the treatment of allergic asthma and other allergic diseases, but their usefulness is limited by side effects. Drugs that inhibit effector cytokines, but lack the side effects of glucocorticoids, would potentially be very useful in the treatment of allergy. Our findings suggest that, when such novel drugs are evaluated, their effects on IL-9 should be taken into consideration.

## Competing interests

The author(s) declare that they have no competing interests.

## Authors' contributions

LEH performed the RT-PCR experiments. KPJ performed the ELISA experiments. LEH and KPJ drafted the manuscript. JvS prepared the anti-IL-9 antibodies and revised the manuscript. FC prepared the anti-IL-9 antibodies. WAS conceived of the project, supervised its design and coordination, and revised the manuscript. All authors read and approved the final manuscript.
